# Membranous Dysmenorrhœa

**Published:** 1875-08

**Authors:** Robert Battey

**Affiliations:** Atlanta, Ga.


ATLANTA

MEDICAL AND SURGICAL JOURNAL.

VOL. XIII.] AUGUST—1875. [No. 5.

### Original Communications.

### MEMBRANOUS DYSMENORRHŒA.

BY ROBERT BATTEY, M.D., ATLANTA, GA.

*Read before the Academy of Medicine June 29th, 1875.*

It will be recollected that I presented to the Academy a few weeks ago an entire cast of the uterine cavity, which I believed to be a true dysmenorrhœal membrane, but which had been so long macerated in water as to render it unfit for microscopical examination, as reported by Dr. Baird, to whom the specimen was referred. More recently I have procured another specimen from the same patient, which, however, is broken into fragments, and it has been put into the hands of Drs. Baird and Simpson for examination and report. I now propose to give a brief history of the case, and indulge in some scattered remarks touching the general subject of membranous dysmenorrhœa.

Miss. Jenny, a single lady, aged thirty, began her menstrual life at the age of fifteen. For six years past she has been a martyr to dysmenorrhœa, and has passed at every period a dysmenorrhœal membrane, usually whole, as a distinct cast of the uterine cavity, being hollow within and presenting the three openings, one for the os and two for the fallopian tubes. This membrane is passed after great and prolonged uterine pain. Occasionally, but rarely, the membrane is discharged in pieces, as upon the last occasion, and the pain is then greatly lessened in degree, or may be comparatively absent.

For twelve months past she has been under my observation, and for the greater part under my professional care. She has been treated by sponge tents and intra-uterine applications of nitrate of silver, carbolic acid, iodine, etc. The use of the tent has given her marked relief of pain, by enlarging the cervical canal just before the periods and permitting the more easy passage of the membranes, but no influence whatever has been exerted over the regular monthly formation of the membrane. Her general health is so broken down by her sufferings that she has been for years confined to the house, and for a week at each period to her bed. Life is shorn of its pleasures, and she spends three consecutive weeks in dread contemplation of the inevitable sufferings of the fourth, in each recurring lunar month. What is to be done for her relief?

To arrive at a proper understanding of this form of disease it is important first to determine what is properly meant by the term membranous dysmenorrhœa. Thomas gives us a definition which seems clear, practical and satisfactory, namely: "This variety of dysmenorrhœa consists in the expulsion of organized material from the uterine cavity, at menstrual periods, which is found upon microscopical examination to consist of the lining membrane of the uterus itself. This may consist of a sac, representing the triangular cavity of the body of the uterus with its three openings, or it may come away piecemeal as shreds or strips of mucous membrane." To determine the fact that it is the true mucous lining or endometrium, it is of course necessary that the specimen shall present the proper histological characters. If we admit under the term dysmenorrhœal membrane every stringy, or fleshy, or fibrinous mass which may chance to pass from the uterus at a menstrual period, we shall inevitably be led into the greatest confusion, *e.g.*, it is a familiar fact that blood clots may be retained in the cavity of the uterus, or in the vaginal cul-de-sac, and, becoming washed continuously by the secretions and deprived in great part of red globules, assume a fleshy or membranous appearance well calculated to deceive the naked eye.

It would appear from the dictum of authorities, which accords with my own observation, that this form of dysmenorrhœa is not of frequent occurrence. Dr. Gaillard Thomas states, in his last work, that he has encountered it but five times; Barnes regards it as very uncommon in single women; Byford pronounces it more rare than other forms of dysmenorrhœa; Hewitt speaks of it as very infrequent.

The pathology and etiology of membranous dysmenorrhœa has been the subject of much discussion and diversity of opinion, and is still far from settled. There is, however, one point upon which the mind of the profession may be said to be fixed—*i.e.*, the uterine cast thrown off at the menstrual period is not the fibrinous detritus of a macerated blood-clot—is not an early abortion—is not a diphtheritic membranous exudation of the endometrium—is not an exfoliation of vaginal tissue, but *is* the veritable lining of the uterine cavity itself.

The cause of this exfoliation of the endometrium is still a matter of dispute. Sir James Simpson, who gave much study to the subject says: "I have always taught that we have here to do, not with an inflammatory exudation, but with a desquamation or exfoliation of the mucous membrane of the uterus; and the proof that such is the case is irresistible. For if you examine it, particularly when it is placed in water and unfolded, you will find it to resemble, in every respect, the early decidua. Like the decidua, it is a shut sac, triangular in form, rough and irregular on the outer surface, and on the interior smooth and of a cribriform appearance. It possesses the complex structure of the mucous membrane of the uterus, containing crypts or follicles with nucleated cells or vessels intervening; and in all respects corresponds to the mucous membrane, as it presents itself to us more especially in that hypertrophied condition which is seen in the earlier periods of pregnancy." He regards the exfoliation as essentially non-inflammatory, but merely an exaggeration of a normal condition, or an exalted degree of a physiological action. Scanzoni attributes the phenomenon to "a considerable hyperæmia of the walls of the uterus, which is followed by an excess in the development of the mucous membrane." Klob says that "those pathologists were not far from the truth who described such cases as endometritis." Some have gone so far as to assert the absolute identity of this membrane with the decidua, and to claim that it can only occur in the subjects of married life, and as the product of conception. Others deny this assertion, but, admitting the similarity, use the term "menstrual decidua," and attribute its formation to an abnormal ovarian influence. We learn from Barnes that Courty actually extracted with forceps from the os uteri of a virgin the membrane through a small speculum manipulated so as not to break down the hymen. Whilst membranous dysmenorrhœa certainly occurs most frequently in the married, its occurrence likewise in virgins cannot be denied.

THE SYMPTOMS of the disease, as presented in the case under consideration, are first, intense pain in the hypogastrium, hips and back, commencing shortly after the first show of the menses; secondly, arrest of the flow during the existence of these pains, which last from one to three days; thirdly, discharge of the membrane with relief to the pains and return of the flow, which becomes excessive and lasts for eight days from the beginning. The pain suffered is often excruciating; it is expulsive in character, and comparable to the pains of abortion.

Of the PROGNOSIS of this disease there is little that I can say from my own observation of the results of treatment. Very few cases have ever passed through my hands, and I have thus far nothing encouraging to report in the way of permanent cure.

Well do I now remember when, after some years of pharmaceutical study and practice at the dispensing counter, interspersed with much unprofitable reading of patent medicine almanacs, circular, and certificates of marvelous cures, when I came forth from the portals of my medical *alma mater* with my parchment roll under my arm, filled to my humble capacity with the self-satisfying lore of the schools, recounting in advance the long series of glorious conquests over disease and death which should marshall the way to future fame and fortune. What a happy day! How easy the problems before me! What stupendous results awaited me! With what a softened and compassionate heart did I regard my seniors, who battled disease with stores of common sense and long experience, but who lacked the essential palladium from the sheep's pelt! How simple were the problems of disease; how resistless the logic of the materia medica! From that day to this my professional career has been full of lessons of distrust of drugs and reliance upon good sense, as the handmaid of nature, in the cure of disease.

In the department of gynæcology, too, I would fain make confession of my shortcomings. Formerly the speculum, the tent, the nitrate pencil and other local medicants possessed a talismanic power over intra-uterine chronicities, and I numbered my triumphant cures by scores and scores, amongst whom very many were either deceived like myself, or willing to abet my own self-deception to rid themselves of an irksome, protracted and expensive course of treatment. Many, doubtless, believed themselves to be cured until time, the wise schoolmaster, undeceived them. I too have unlearned in the school of experience much of my former knowledge upon this subject, and find myself to-day an humble inquirer, groping my way darkly in the midst of numerous much vaunted panaceas, seeking to lay hold upon some hidden truth, upon which I may venture to place a foot with some assurance that the next ebbing of the tide will not sweep it away from me.

Thomas says of this disorder: "The prognosis is extremely unfavorable." Barnes says: "But with all possible care we must be prepared to find these cases rebellious to treatment for a long time." Byford says: "The membranous form is very difficult of cure, and can seldom be accomplished with the ordinary modes of treatment." Hewitt says: "The absolute cure of cases coming under this category is a problem which yet requires solution, and the fact that the subjects of it are generally sterile, and not unfrequently extremely desirous of children, renders this question additionally interesting." West says: "When this condition has existed for years, it becomes, I fear, almost incurable." Scanzoni says: "According to our experience, the least favorable cases are those where the dysmenorrhœa is accompanied with the expulsion of the membranes which we have described, and where the malady is due partly, perhaps, to the presence of these membranes. At least, in such like cases, we have never obtained a complete cure; others claim to have been more successful."

For the Treatment of the disease little need be said. The management of the paroxysm is palliative, and is conducted upon the general principles which obtain in other forms of dysmenorrhœa. As regards the curative treatment, I find no evidence of the *cure* of the disease which is not clouded by great uncertainty in regard to the accuracy of the differential diagnosis, and the malady may be fairly classed amongst the *opprobria medicorum* until science shall have shed new light upon it. It is at present, I believe, only to the *change of life* that we are to look for the cure of true membranous dysmenorrhœa; whether this change of life be effected in the accustomed order of nature, or by art, in the operation of Normal Ovariotomy.

